# Investigating Mechanical Properties of Alkali-Activated Slag Cementitious Material for Load-Bearing Layer of Sandwich Panels

**DOI:** 10.3390/ma16196398

**Published:** 2023-09-25

**Authors:** Jing Zhu, Zijian Qu, Ying Huang, Lizhuo Song, Shaotong Liu, Hao Min, Zhiming Li

**Affiliations:** 1 College of Civil Engineering and Architecture, Harbin University of Science and Technology, Harbin 150080, China; 1715020417@stu.hrbust.edu.cn (Z.Q.); songlizhuo12@163.com (L.S.); liushaotong1998@163.com (S.L.); meehyy@foxmail.com (H.M.); lizhiming0506@163.com (Z.L.); 2 Department of Civil Engineering, North Dakota State University (NDSU), Fargo, ND 58102, USA

**Keywords:** fiber reinforcement, alkali-activated slag cementitious materials, sandwich panels, mechanical properties

## Abstract

The research presented in this paper is about the mechanical properties of fiber-reinforced alkali-activated slag cementitious sandwich panels with different types and amounts of admixtures. The mechanical properties, drying shrinkage properties, and micro-morphology were used to determine the optimal ratio of the admixtures. The results show that the alkali-activated slag sandwich panels have the characteristics of light weight, high strength and excellent thermal insulation, and the factors such as magnesium oxide, expansion agent and solution temperature have significant influence on their mechanical properties and dry shrinkage. This paper provides a theoretical basis and experimental data for the preparation process and application of alkali-activated slag sandwich panels.

## 1. Introduction

Sandwich-type insulation wall panels have been extensively studied for their light weight and thermal insulation properties. They have been found to be widely used in exterior wall insulation systems worldwide. This thesis focuses on the load-bearing layer of sandwich panels, which is produced using alkali-activated slag cementitious material (AASCM) [1,2,3]. Originating from the work of Professor Glukhovsky in 1957, AASCM is formulated by activating industrial waste residues, such as slag, using alkaline activators [4]. These materials have achieved compressive strengths of up to 120 MPa and have demonstrated remarkable stability [5,6,7].

Sandwich-type insulation wall panels has been a subject of extensive research since the 1960s, particularly as external wall insulation systems. Recent advancements have highlighted the unique advantages of lightweight, high-strength, and superior thermal insulation properties. This has led to a surge of interest in fiber-reinforced AASCM sandwich panels [8,9]. Liu et al. [10] conducted double shear tests on concrete sandwich panel specimens reinforced with basalt fiber-reinforced composite (BFRP) connectors, deriving a bearing capacity formula. Tomlinson et al. [11] developed a numerical model for predicting the response of partially composite load-bearing concrete sandwich panels under arbitrary eccentric loads. Alchaar et al. [12] performed a finite element analysis, demonstrating that the composite action of the sandwich panel led to ductile failure rather than sudden fracture.

As a new environmentally friendly building material, the field of AASCM had been rigorously studied, with a focus on mechanical properties and durability. Mavroulidou et al. [13] delved into the role of different activators, comparing sodium carbonate-activated slag and sodium silicate-activated slag. Kiachehr et al. [14] explored the effects of curing time and temperatures on alkali-activated concrete, proposing a predictive model for residual strength and mass loss. Mikhailova et al. [15] investigated the influence of shrinkage-reducing agents like polyethylene glycol and polypropylene glycol, resulting in a model for estimating shrinkage. Aydın et al. [16] specifically studied the drying shrinkage of AASCM using steel fiber-reinforced alkali-activated slag/silica fume mortar and proposed a drying shrinkage stress model.

While AASCM has shown promising mechanical properties, its substantial drying shrinkage has limited its broader applications. This issue is particularly relevant when considering the load-bearing layers of sandwich panels. The existing literature lacks comprehensive studies on the effects of different admixtures and solution temperatures on AASCM’s mechanical properties and drying shrinkage. Furthermore, the impact of additives on the microscopic morphology of AASCM is not well-understood, leaving room for further exploration.

This study focuses on a comprehensive investigation into the mechanical properties and drying shrinkage of AASCM, in order to address existing gaps in the literature. Specifically, the research will explore the effects of different admixtures and varying solution temperatures on these properties when AASCM is used in the load-bearing layer of sandwich panels. Additionally, the study will examine the impact of additives on the microscopic morphology of AASCM. By achieving these objectives, the research will contribute to a deeper understanding of how admixtures and solution temperatures can optimize the material for use in the load-bearing layers of sandwich panels, thereby expanding their potential applications.

## 2. Material Proportions and Experimental Design

### 2.1. Materials

AASCM is an early-strength material. To apply this material on a large scale, it is necessary to reduce the hydration heat and microcracks generated by the intense early hydration reaction. Therefore, aggregates, gypsum, fly ash, silica fume, etc., were added to the material to lower the early reaction degree of the AASCM. MgO and expansive agents were added to compensate for shrinkage and inhibit crack formation. Specifically, to optimize the proportion of AASCM for balanced mechanical and drying shrinkage properties, these different types of admixtures using different amounts were selected to be investigated in this study. The materials used in this study included cement, slag, potassium silicate, magnesium oxide, refractory fiber, and other reagents, and their treatment and mix design are detailed in this section.

#### 2.1.1. Slag

**Figure 1:**
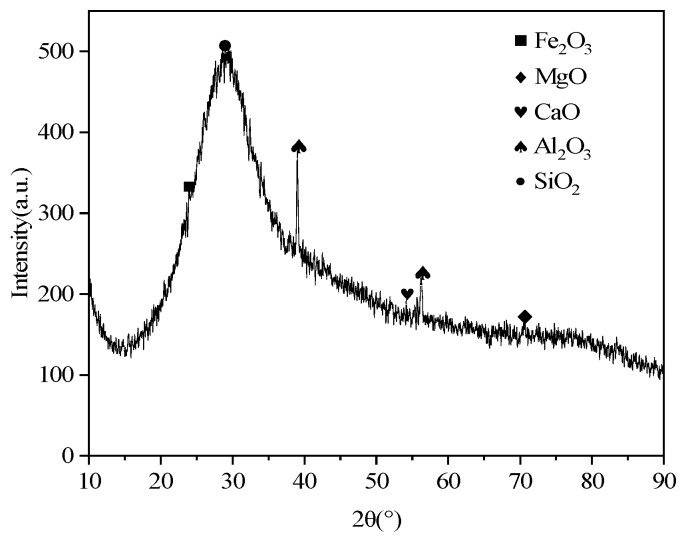
XRD pattern of slag.

The S105-grade slag used in this study was an industrial byproduct generated during the iron reduction process at Tangshan Iron Orchid Company (Hebei, China). The slag had an alkalinity coefficient, M_0_ = (MgO% + CaO%)/(SiO_2_% + Al_2_O_3_%), of 0.87, indicating that it was acidic slag. Its specific surface area was 550 m^2^/kg. The slag’s chemical composition and XRD pattern are shown in Table 1 and Figure 1, respectively. As can be seen in Figure 1, the bread loaf-shaped peak represents amorphous SiO_2_, with almost no distinct characteristic peaks, indicating that the slag primarily exists in an amorphous glassy state.

#### 2.1.2. Potassium Silicate

**Figure 2:**
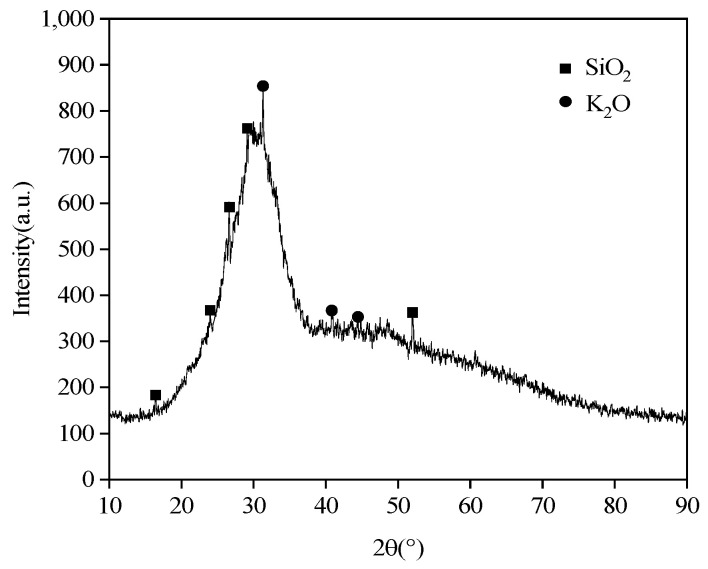
XRD pattern of potassium silicate.

The potassium silicate used in the tests described in this paper was provided by Xingtai Da Yang Chemical Co., Ltd. (Xingtai, China). The XRD pattern shows that the main components of the dried potassium silicate powder are SiO_2_ and K_2_O in Figure 2. Combined with the production information, it can be learned that the chemical formula of potassium silicate is K_2_O·n SiO_2_·H_2_O, the initial modulus is 2.78, the group degree is 46.3, the content of silica is 27.49%, the content of potassium oxide is 15.50% and the density is 1.434 g/mL.

#### 2.1.3. Magnesium Oxide

**Figure 3:**
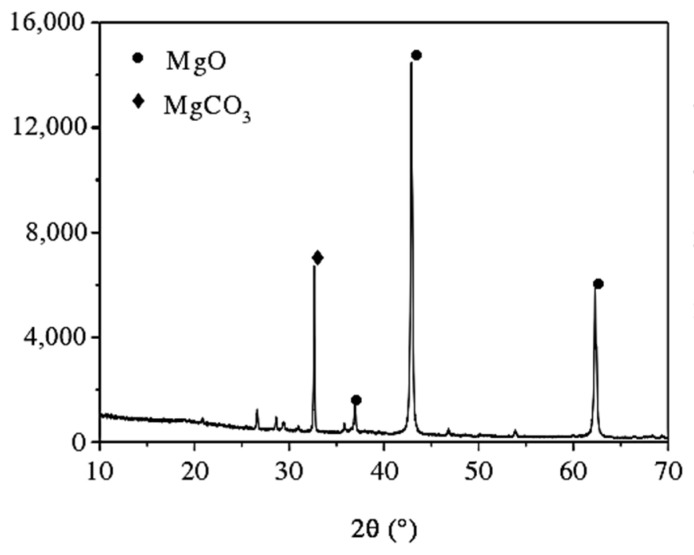
XRD pattern of magnesium oxide when lightly burned.

Lightly calcined magnesium oxide powder was produced by Liaoning Dashiqiao Tianyi Refractory Materials Co., Ltd. (Dashiqiao, China). It was processed from magnesite ore calcined at 950 °C. The chemical composition and XRD pattern of lightly calcined magnesium oxide are shown in Table 2 and Figure 3, respectively. The active content determined using the hydration method was 52.3%.

#### 2.1.4. Refractory Fiber

The refractory fiber was sourced from Shandong Luyang Energy Saving Materials Co., Ltd. (Zibo, China). It was a polycrystalline refractory fiber that included mullite fiber, alumina fiber, and zirconia fiber, with single filament diameters of 3 to 4 μm.

#### 2.1.5. Other Reagents

Expansive agent: The applied expansive agent was from Henan Mingzhu Chemical Co., Ltd. (Puyang, China), with the main component being calcium oxide (CaO) at a content of 70.8%.

Gypsum: Desulfurized gypsum was used in this study with its main component being calcium sulfate dihydrate (CaSO_4_·2H_2_O) at a content of ≥93%. Processed desulfurized gypsum is a typical environmentally friendly material.

Fly ash: The fly ash used in this study was provided by Lingshou Chuangbo Mineral Products Co., Ltd. (Shijiazhuang, China). Fly ash consists of fine ash particles discharged during the combustion of fuel (mainly coal). Its particle size generally ranges from 1 to 100 μm. Also known as pulverized coal ash or soot, it comprises fine solid particles from the smoke and ash generated by fuel combustion.

Silica fume: The silica fume used in this study was obtained from Gansu Lixin Source Micro Silicon Powder Co., Ltd. (Lanzhou, China). Silica fume is produced from the dust emitted with exhaust gases during the high-temperature smelting of industrial silicon and ferrosilicon in an electric furnace. It is collected and treated using specialized equipment. The SiO_2_ content in the emitted dust accounts for approximately 90% of the total dust mass, and the particle size is exceptionally small, with an average size close to the nanometer scale.

Sodium hydroxide: Sodium hydroxide was supplied by Tianjin Tianli Chemical Reagent Co., Ltd. (Tianjin, China), with a mass fraction of ≥96.0%.

Water: Tap water (H_2_O) was used for the experiments in this study.

### 2.2. Mix Ratio Design

#### 2.2.1. Adjusting the Modulus of Potassium Silicate to 1.0

According to previous studies, the optimal modulus of potassium silicate used for the preparation of AASCM was 1.0 [17]. The original potassium silicate had a modulus of 2.78, which meant that the molar ratio in the solution was SiO_2_:K_2_O = 2.781. When the modulus was adjusted to 1.0, the molar ratio in the solution was SiO_2_:(K_2_O + NaOH) = 2.78:(1 + 1.78). Each gram of potassium silicate contained 0.155 g of K_2_O, which was converted to a molar amount of 0.0017 mol. To adjust the modulus to 1.0, the molar amount of NaOH added to each gram of potassium silicate should have been 0.006052 mol, which, when converted to mass, was 0.24208 g.

#### 2.2.2. Treatment of Refractory Fibers

**Figure 4:**
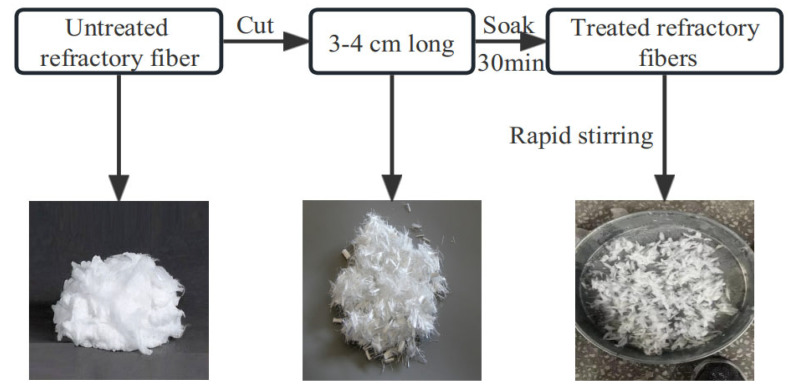
Refractory fiber treatment diagram.

Untreated refractory fibers have a cotton-like appearance, and mixing them directly with AASCM may have resulted in uneven fiber distribution in the specimens. As shown in Figure 4, to better mix the fibers within the AASCM, the fibers were cut into 3–4 cm long pieces using scissors. The chopped fibers were soaked in a sodium potassium silicate solution for 30 min, followed by rapid stirring of the solution. Once the fibers were dispersed in the solution, the solution and fibers were poured into the slag and mixed together.

#### 2.2.3. Mix Ratio Design

In order to study the influence of different admixtures, 12 different mix designs were tested in this study, and the mix design numbers ranged from S-1 to S-12. The material combinations required per cubic meter of AASCMS are shown in Table 3. Previous studies had shown [18] that AASCM exhibits good performance when the modulus of potassium silicate is 1.0, the alkali content is 14% and the fiber content is 1% by mass. Thus, the fiber content in this study adopted 1% for all testing samples. The strength of the AASCM mortar is not only related to the properties of the binder material but also to the water-to-binder ratio and binder-to-sand ratio. Shen et al. [19] found through experiments that a larger water-to-binder ratio results in more capillary pores, while a smaller binder-to-sand ratio reduces cement usage and increases binder–sand interfaces, leading to more interface microcracks, capillary pores and microcracks. Therefore, this study selected a binder-to-sand ratio of 1:2 and a water-to-binder ratio of 0.5 to meet the casting requirements of sandwich panels.

### 2.3. Testing Methods

#### 2.3.1. Compressive and Flexural Strength Tests

The specimens of the compressive and flexural strength testing were prepared following the following steps:

- Add an appropriate amount of sodium hydroxide to the potassium silicate with a modulus of 2.79 and stir well to adjust the modulus to 1.0.
- Pour slag and MgO into the NJ-160A cement mortar mixer according to the proportion, and mix at low speed for 1 min at a speed of 140 r/min to achieve a uniform powder mixture.
- Pour the potassium silicate, water and refractory fibers into the mixer and mix at low speed for 2 min at a speed of 140 r/min, followed by high-speed mixing for 2 min at a speed of 285 r/min.
- Pour the mixed slurry into 40 × 40 × 160 mm prismatic three-gang molds, ensuring the slurry covers the entire mold to prevent size deficiencies in the specimens due to slurry leakage during compaction, which may lead to incorrect flexural and compressive strength results.
- Compact the slurry on a vibrating table 120 times, and then, place the molds in a curing chamber with a standard temperature of 20 ± 2 °C and a relative humidity of 95% for curing. Demold the specimens after 1 day of curing.

**Figure 5:**
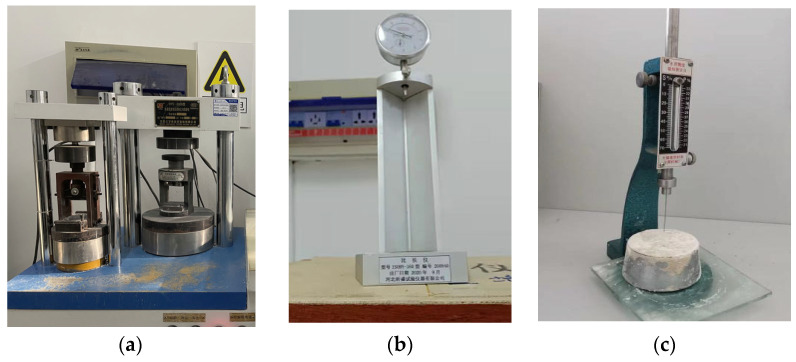
Testing equipment used: (**a**) compressive strength testing machine, (**b**) comparator and (**c**) Vicat apparatus.

The specimens were cured for 3, 7 and 28 days and tested using the HYE-300B cement flexural and compressive constant stress testing machine, as shown in Figure 5a, following testing standard JGJ/T70-2009 “Standard for test method of performance on building mortar” [20]. The flexural strength was tested by applying a load uniformly at a rate of 50 N/s until failure. After the flexural strength test was completed, the specimens were cut into two pieces. Then, the compressive strength of the intact specimens without any other damage was tested at a loading rate of 2400 N/s. The arithmetic mean of the measured values of three specimens was used as the compressive strength of the mortar cubic specimens for the test group.

#### 2.3.2. Drying Shrinkage Test

The drying shrinkage tests were performed using the BC-160-type comparator, as shown in Figure 5b. The comparator was placed on a flat table and adjusted to have the pointer aligned with the “0” on the scale. The clean specimen was placed in the comparator and tested for the shrinkage by observing the dial indicator. Due to mold factors, the length of the specimen could not be exactly 160 mm, so the initial length was determined by the length of the specimen when demolded at 1 day. The drying shrinkage could be estimated from the reading of the drying shrinkage tests as follows:

(1) $$\varepsilon =\frac{L_{1}-L_{t}}{L_{t}}$$

where ε is the shrinkage rate of the specimen at t (3, 7, 28) days; *L*_1_ is the length of the specimen at 1 day, in mm; and *L*_t_ is the length of the specimen at t days, in mm.

#### 2.3.3. Setting Time Test

To prepare the specimens for setting time tests, the well-mixed mortar was pulled into the round mold and placed in the curing chamber for 10 min. The specimens were then removed from the mold and tested using the Vicat apparatus, as shown in Figure 5c. The Vicat apparatus measured the initial setting time every 5 min. To obtain the setting time, the timer was started from the moment water was added and continued until the needle stopped sinking or, after releasing the needle for 30 s, the pointer reading was 4 mm ± 1 mm from the bottom plate. This time was measured as the initial setting time of the mortar.

After determining the initial setting time, the round mold with an inner diameter of 65 mm was rotated immediately by 180° so that the top faced downward, and it was then placed in the curing chamber. As the final setting time approached, measurements were conducted every 15 min or at shorter intervals. The timer was started from the moment water was added and continued until the final setting needle, when approaching the specimen, did not leave a trace on the specimen after freely falling for 30 s. This was measured as the final setting time.

#### 2.3.4. Microstructural Analysis Methods

(1) X-ray Diffractometer (XRD): XRD tests were performed on the samples from the specimens using D8-Advance from Bruker Corporation (Billerica, MA, USA). To prepare the testing samples, the specimens were crushed and grinded into powder and then passed through a 0.075 mm sieve. The powder was dried in a beaker at 40 °C until a constant weight was achieved. The phases were scanned using an XRD instrument with a measurement accuracy of ≤0.010 and the scanning range of 10–70°.
(2) Scanning Electron Microscope (SEM): SEM analyses were performed on the specimens using SUPERTM 55 from Carl Zeiss AG (Oberkochen, Germany). The testing samples were prepared as follows: (a) the broken center parts of the specimens with a curing age of 3 d and 28 d were placed in anhydrous ethanol to stop hydration; (b) after soaking for 7 days, the samples were dried at 60 °C in an oven until a constant weight was achieved; and (c) the samples were then attached to a tray and coated with gold. After gold coating, the SEM analysis was performed.

## 3. Results and Discussions

### 3.1. Effect of Admixture on Mechanical Properties of AASCM

#### 3.1.1. MgO Content

**Figure 6:**
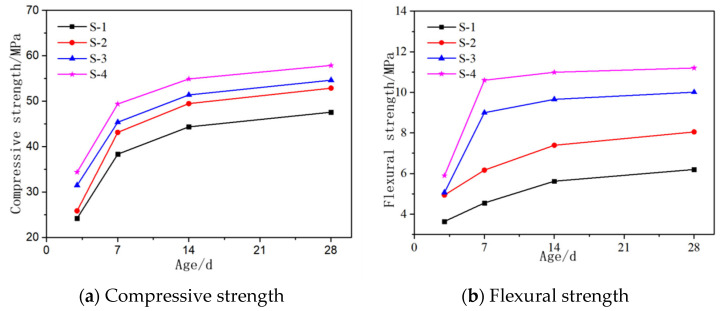
Effect of magnesium oxide content on compressive and flexural resistance of materials.

The influences of MgO content ranging from 0% to 30% at an interval of 10%, as shown in Sample S1–S4 in Table 3, on the compressive and flexural strength of AASCM are shown in Figure 6a,b, respectively. It can be seen that as the MgO content increased, the compressive and flexural strength of the AASCM increased at all ages. MgO had a more significant effect on the flexural strength than on the compressive strength of AASCM, and it improved the low early flexural strength of AASCM. The MgO content improved the strength of AASCM because after hydration, MgO formed brucite (Ht) and magnesium silicate hydrate gel [21] (M-S-H), releasing OH-ions into the solution. The high concentration of OH^-^ ions entered the slag glass network, destroying the network structure and accelerating the dissolution of the slag glass. The rapid dissolution of slag glass led to a high concentration of Ca^2+^, Mg^2+^ and SiO_4_^4−^ in the solution, accelerating the formation of calcium silicate hydrate gel (C-S-H), brucite and magnesium silicate hydrate gel.

In addition, a greater increase in the early curing stages was noticed when compared to the later curing stages. The smaller strength increase in the later curing stages may have been due to the decreasing ion concentration in the later curing stage, which slowed the reaction rate. Furthermore, there was a large amount of Mg^2+^ ions near the slag particles, which reacted with the SiO_4_^4−^ ions dissociated from the potassium glass in water to form M-S-H, encasing unreacted slag particles and hindering the dissolution of slag [22].

**Figure 7:**
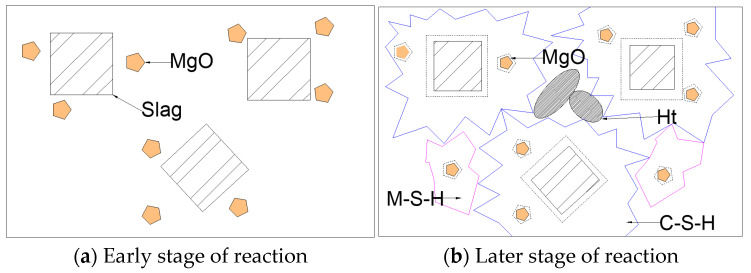
Reaction mechanism diagram of magnesium oxide in AASCM.

The more pronounced effect of MgO on the flexural strength of AASCM may have been due to the fact that the density of both Ht and M-S-H generated by the hydration of MgO was smaller than that of C-S-H, which could more effectively fill the pores between materials [23], as shown in Figure 7. The filling of pores made AASCM more compact, and a smaller porosity led to a more significant increase in compressive strength.

#### 3.1.2. Partial Replacement of MgO with CaO

**Figure 8:**
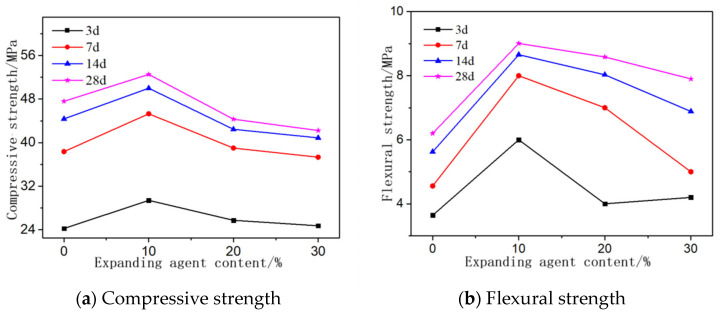
Effect of expansion agent content on compressive and flexural resistance of materials.

The influences of the partial replacement of MgO with CaO on the compressive and flexural strength of AASCM are shown in Figure 8a,b. The highest compressive and flexural strength of the AASCM was noticed to be with a CaO content of 10%. After the addition of 10% CaO, the further increase in the CaO content decreased the compressive and flexural strength of the AASCM. Such an optimal ratio of CaO replacement ratio might have been induced by the fact that CaO hydrolyzes faster than MgO, so the added CaO could rapidly hydrolyze to form Ca(OH)_2_. As Ca(OH)_2_ continuously dissociated Ca^2+^ and OH^-^ into the solution, the high concentration of OH^-^ was beneficial for the dissolution of slag. When the CaO content was low, the Ca/Si molar ratio in the solution was low, and there was a large amount of free SiO_4_^4−^ in the solution. The faster hydration rate of CaO resulted in a higher pH value in the early stage, which was beneficial for the hydration of Mg^2+^ to form Ht and M-S-H, thereby increasing the strength of the material. When the CaO content was higher than 10%, the Ca/Si molar ratio increased, and SiO_4_^4−^ preferentially reacted with Ca^2+^ to form C-S-H, inhibiting the reaction between SiO_4_^4−^ and Mg^2+^ and hindering the formation of Ht and M-S-H [24]. Moreover, as the CaO content increased, the concentration of Ca^2+^ increased, and there was not enough free SiO_4_^4−^ in the solution to form M-S-H. Mg^2+^ existed in the form of Mg(OH)_2_, which affected the strength of the material.

#### 3.1.3. Other Admixtures

**Figure 9:**
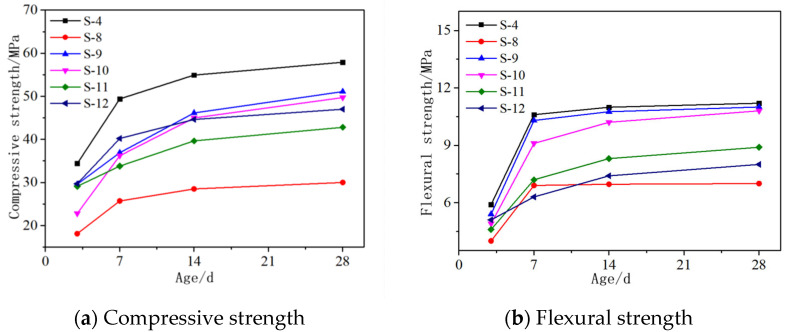
Effect diagram of other admixtures on compressive and flexural resistance of materials.

The related literature [25] shows that gypsum, fly ash, cement and silica fume have a beneficial effect on the mechanical properties of AASCM. Therefore, this study set the gypsum content at 12%, fly ash content at 8%, cement content at 8% and silica fume content at 2%. Data obtained from different combinations are shown in Figure 9a,b. It can be seen that when MgO was added alone, AASCM had the best compressive and flexural strength. When MgO and gypsum were added simultaneously, the strength was lower than when MgO and gypsum were added separately. Adding gypsum and fly ash will reduce the early strength of AASCM, while adding cement and MgO will increase the early strength of the AASCM. Since MgO has an activating effect on AASCM, the strength was higher than that in other groups. Gypsum and fly ash did not show as high reactivity as MgO in the material and are non-reactive or weakly reactive items, hindering the hydration of slag.

### 3.2. The Influence of Solution Temperature

When casting large components, the hydration heat accumulated inside the member is not easily dissipated, leading to potential for the internal hydration heat to reach 50 °C to 60 °C. The temperature stress causes expansion cracks in the components. Thus, measures are needed to reduce the hydration heat for AASCM when used on a large scale. Ma et al. [26] studied the strength development of alkali-activated slag mortar under ambient and high temperatures and found that heat-cured samples rapidly gained strength, but if given enough time, ambient temperature curing could produce comparable strength. Therefore, this study used the heat released by NaOH to dissolve in the solution and divide the solution temperature into four different temperature stages based on time, as shown in Table 4. Specimens were prepared at time t = 5 min, 30 min, 60 min and 1 day (1 d) to study the influence of NaOH dissolution time on the mechanical properties and workability of the material.

**Figure 10:**
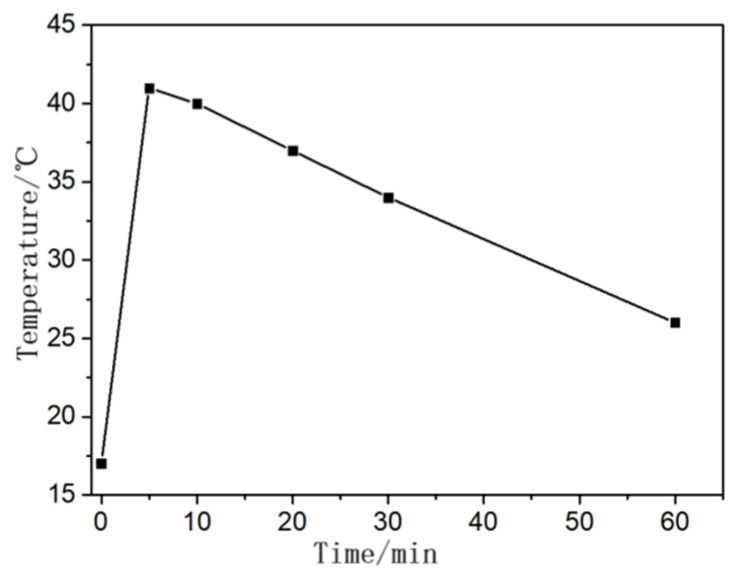
Temperature over time.

To measure the temperature change for NaOH dissolution in water, the air temperature was measured using a thermometer, which was 18 °C, in addition to water temperature, which was 17 °C. Then, the weighed NaOH was poured into a potassium glass to adjust the modulus and the thermometer was placed in the NaOH solution to measure temperature stages with different NaOH dissolution times. The temperature was read and recorded at intervals, as shown in Figure 10. Figure 10 indicates that a large amount of heat was released when NaOH dissolved, and the solution temperature rose to 41 °C in 5 min. Afterward, the temperature began to decrease, with the early stage decreasing slower than the later stage as the NaOH in the solution was still dissolving. The solution temperature was the same as the room temperature after 1 day.

**Figure 11:**
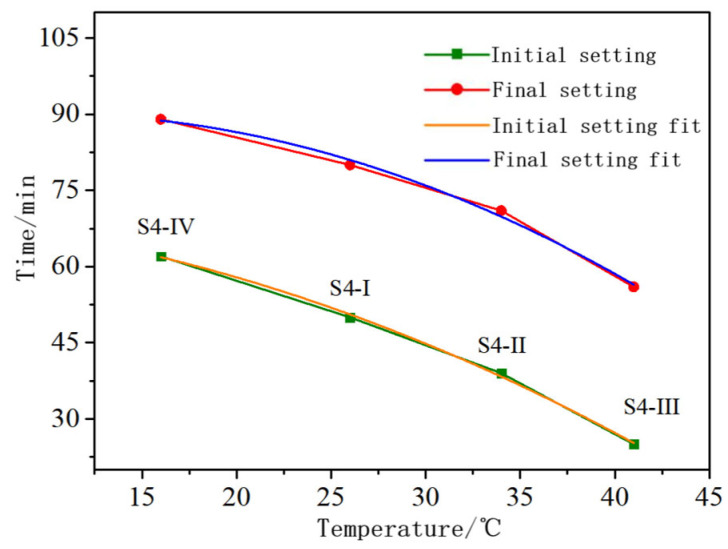
Diagram of the relationship between solution temperature and initial and final setting time.

Figure 11 plots the relationship between the solution temperature and the initial and final setting times. Both the initial and final setting times increased with the increase in solution temperature; however, the setting time between the initial and final setting did not increase significantly. This indicates that the temperature of the solution has a significant impact on the setting time. The setting time decreased with the increase in temperature. The setting time and temperature showed a good quadratic polynomial fitting relationship, and the significance of the relationship can be determined by *R*^2^ as:

(2) $$\begin{array}{ll} T_{1}=70.59-0.188x-0.022x^{2} & R^{2}=0.996\\ T_{2}=86.82+0.67x-0.034x^{2} & R^{2}=0.987 \end{array}$$

in which *T*_1_ is the initial setting time, in minutes; *T*_2_ is the final setting time, in minutes; *x* is the temperature, in °C; and *R*^2^ is the correlation coefficient, with values closer to 1 indicating a higher degree of fit.

The increase in solution temperature accelerates ion movement, which in turn accelerates the decomposition of slag glass and magnesium oxide. The increases in Mg^2+^, Ca^2+^ and SiO_3_^2−^ concentrations in the solution accelerate the formation of C-S-H gel, Ht and M-S-H gel [27]. Therefore, the higher the temperature, the shorter the setting time of the material.

**Figure 12:**
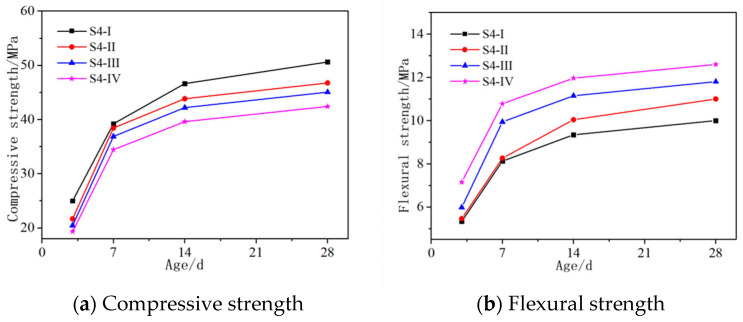
Effect of solution temperature on AASCM compressive and flexural strength.

Figure 12a,b show the influence of solution temperature on the compressive and flexural strength of AASCM. It can be seen that the compressive strength decreased with the increase in NaOH dissolution time, while the flexural strength showed the opposite trend. This phenomenon may be accounted for by the fact that at higher temperatures, more Mg^2+^ is hydrated to form Ht, which is particulate and can fill the pores between C-S-H gels, thereby improving its compressive strength. However, excessive Ht particles may cause gaps in the gel phase and reduce flexural strength. At lower temperatures, more Mg^2+^ is hydrated to form M-S-H gel, which has a smaller density than C-S-H gel [28]. The M-S-H gel formed covers the C-S-H gel, making the structure denser, thereby increasing flexural strength. However, due to the lack of Ht particles to bear pressure, the compressive strength decreases.

### 3.3. The Influence of Admixtures and Solution Temperature on the Drying Shrinkage of AASCM

**Figure 13:**
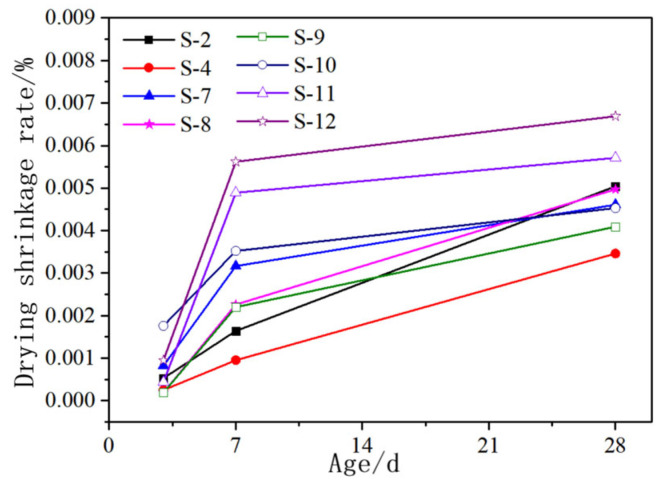
Effect of various admixtures on drying shrinkage of AASCM.

Figure 13 illustrates the effects of various admixtures on the drying shrinkage of AASCM. S-4 has less drying shrinkage than S-2, indicating that increasing the content of magnesium oxide is beneficial for reducing shrinkage. S-4 has less drying shrinkage than S-7, S-8 and S-9, indicating that magnesium oxide is more effective in reducing drying shrinkage than the expansive agents, gypsum and fly ash.

The early hydration of magnesium oxide forms Mg(OH)_2_ has expansive properties to compensate for shrinkage. As the reaction proceeded, Mg(OH)_2_ reacted with SiO_4_^4−^ to form talc and hydrated magnesium silicate. The improvement in material drying shrinkage in the later stage was mainly attributed to the filling of voids by Ht [29]. Therefore, S-4 with 30% magnesium oxide content had less drying shrinkage than S-2 with 10% magnesium oxide content.

The improvement in material drying shrinkage via the expansive agent was mainly due to the formation of Ca(OH)_2_ from some CaO, which acted as an expansive source to compensate for shrinkage [30]. The improvement in material drying shrinkage via fly ash was due to its filler effect, which reduced the content of reactants and reaction activity, resulting in a decrease in mechanical properties. The shrinkage reduction effect was more obvious when a larger amount of fly ash was added [31]. The improvement in material drying shrinkage via gypsum was due to the production of gypsum dihydrate, which can combine with a large amount of water to form a larger solid phase, and the formation of larger-sized dihydrate gypsum.

**Figure 14:**
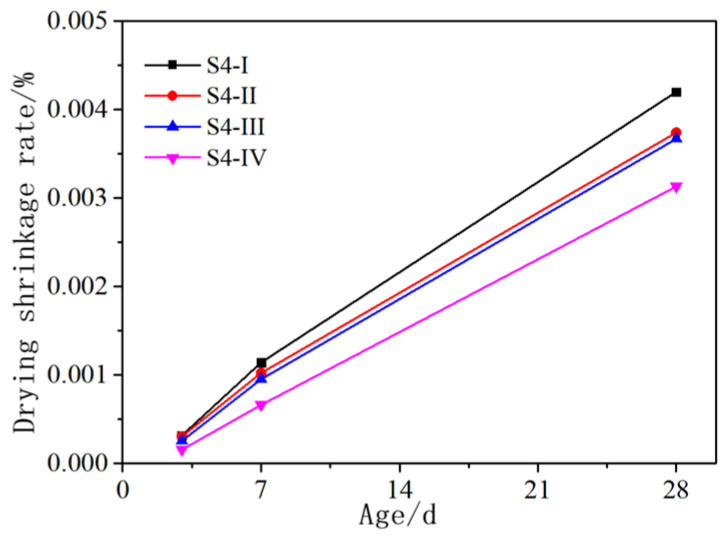
Effect of solution temperature on drying shrinkage of AASCM.

The influence of solution temperature on the drying shrinkage of AASCM is shown in Figure 14. The drying shrinkage was found to decrease with the decrease in solution temperature. This is because the higher the solution temperature, the higher the ion concentration in the early stage of the test, and the early hydration products Mg(OH)_2_ and Ca(OH)_2_ were more abundant. As the reaction proceeded, Mg(OH)_2_ and Ca(OH)_2_ were converted into denser C-S-H and M-S-H, resulting in volume shrinkage. Therefore, the S4-I group had larger drying shrinkage due to the formation of more gel phases.

### 3.4. Microscopic Analysis

#### 3.4.1. X-ray Diffraction Analysis

**Figure 15:**
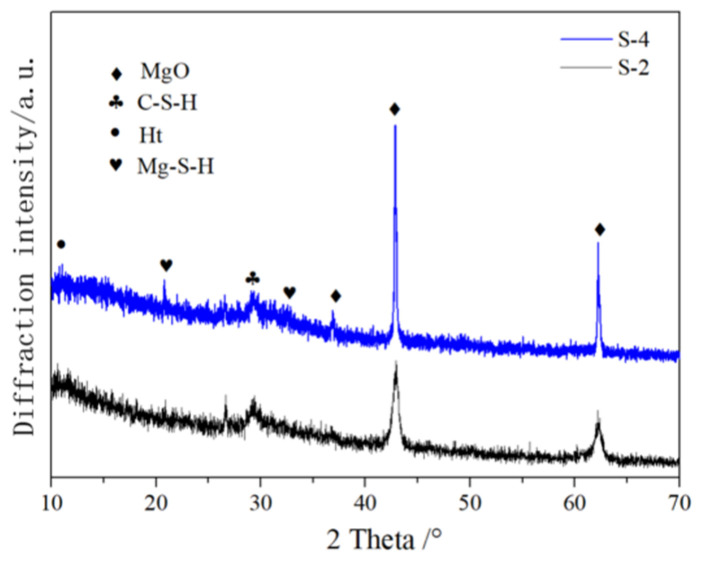
XRD pattern of different magnesium oxide content levels.

The XRD patterns of S-4 and S-2, with 30% and 10% magnesium oxide content, respectively, are shown in Figure 15. From the XRD spectra, it can be seen that at the curing age of 28 days, the unreacted magnesium oxide in AASCM increased with the increase in magnesium oxide content. The Ht peak near 10° also increased with the increase in magnesium oxide content, indicating that magnesium oxide promoted the formation of Ht. The C-S-H peak near 30° became narrower with the increase in magnesium oxide content, and the possible reason for this is that M-S-H covered C-S-H, causing changes in the C-S-H structure.

#### 3.4.2. SEM Analysis

**Figure 16:**
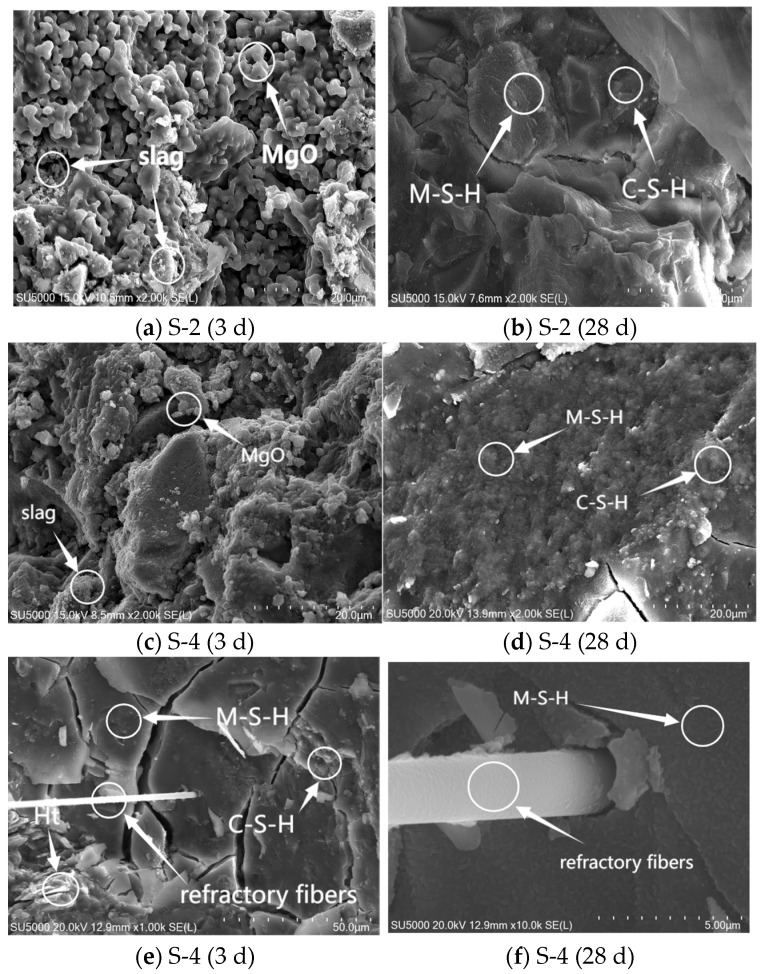
SEM pattern of different magnesium oxide content.

Figure 16a–f show the SEM images of S-4 and S-2 at 3 d and 28 d, with 30% and 10% magnesium oxide content, respectively. From Figure 16a,c, it can be seen that at 3 days, there was still a large amount of unreacted slag and MgO in the system, forming a honeycomb-like porous structure. At 28 days, the hydration of the slurry was more sufficient, making the microstructure denser. From Figure 16b,d, it can be seen that the microstructure density of 10% MgO content was not as good as that of 30% MgO content, which is because the M-S-H formed by MgO could cover C-S-H and compensate for the structural pores [32]. From Figure 16e,f, it can be seen that the refractory fibers had good contact with the matrix, and the fibers were wrapped in dense hydration products. Based on the density and the mottled particles on the surface, it is possible that M-S-H binds C-S-H and Ht and other hydration products together.

## 4. Conclusions

This study provides a theoretical basis and experimental data for the preparation process and application of AASCM sandwich panels. The study also suggests some ways to optimize the mix ratio and curing conditions of AASCM to achieve better performance. The study’s conclusions are summarized as follows:

Magnesium oxide is the most effective admixture for improving the mechanical properties and reducing the drying shrinkage of AASCM. The optimal content of magnesium oxide is 30% by mass of slag.

Solution temperature has a significant impact on the setting time, strength and shrinkage of AASCM. A lower solution temperature leads to a longer setting time, lower compressive strength, higher flexural strength and lower shrinkage.

The microstructure of AASCM is affected by the admixtures and solution temperature. Magnesium oxide promotes the formation of magnesium silicate hydrate (M-S-H) gel and brucite (Ht), which cover calcium silicate hydrate (C-S-H) gel and fill the pores between materials. A higher solution temperature accelerates the hydration of slag and magnesium oxide, resulting in more gel phases and volume shrinkage.

AASCM with the best mix ratio will be used in the research of the sandwich panels, and it will be the main material of the load-bearing layer of sandwich panels in the future.

## Figures and Tables

**Table 1:** Chemical composition of slag.

Component	SiO_2_	Al_2_O_3_	CaO	MgO	Fe_2_O_3_	SO_2_	Others	Loss on Ignition
Content (%)	36.9	15.66	37.57	9.3	0.36	-	0.57	-

**Table 2:** The chemical composition of lightly burned magnesium oxide.

Component	SiO_2_	Al_2_O_3_	CaO	MgO	Fe_2_O_3_	SO_2_	Others	Loss on Ignition
Content (%)	7.32	0.13	2.02	88.36	0.28	-	0.01	1.88

**Table 3:** Mix ratio design.

Mix Design Number	Slag (kg)	Magnesium Oxide (kg)	Expanding Agent (kg)	Gypsum (kg)	Fly Ash (kg)	Cement (kg)	Silica Fume (kg)	Fiber (%)
S-1	1953	/	/	/	/	/	/	1
S-2	1758	195	/	/	/	/	/	1
S-3	1563	391	/	/	/	/	/	1
S-4	1367	586	/	/	/	/	/	1
S-5	1367	/	586	/	/	/	/	1
S-6	1367	195	391	/	/	/	/	1
S-7	1367	391	195	/	/	/	/	1
S-8	1133	586	/	234	/	/	/	1
S-9	1219	586	/	/	156	/	/	1
S-10	1719	/	/	234	/	/	/	1
S-11	1563	/	/	234	/	156	/	1
S-12	1758	/	/	/	156	/	39	1

**Table 4:** Dissolution time selection.

Serial Number	S4-I	S4-II	S4-III	S4-IV
NaOH Dissolution Time	5 min	30 min	60 min	1 d

## Data Availability

Not applicable.
